# Chitosan supplementation reduces oxidative stress in *Leiothrix lutea* in acute heat stress

**DOI:** 10.1002/vms3.387

**Published:** 2020-10-29

**Authors:** Yi Dai, Ming‐qiang Zhou, Yun‐qian He, Xi Peng, Shi‐bin Yuan

**Affiliations:** ^1^ College of Life Sciences China West Normal University Sichuan China; ^2^ Key Laboratory of Southwest China Wildlife Resource Conservation of the Ministry of Education China West Normal University Sichuan China

**Keywords:** chitosan, heat stress, histological change, *Leiothrix lutea*, liver

## Abstract

In order to assess the effects of chitosan supplementation on immune function, antioxidant enzyme activities and histological changes in *Leiothrix lutea* exposed to acute heat stress, 80 healthy adult birds were randomly divided into five experimental groups. The normal‐temperature group (NTG) was maintained at 21°C and fed the basic diet. The treatment groups were fed the basic diet supplemented with 0%, 0.1%, 0.5% and 1.0% chitosan, respectively, in normal‐temperature environment for 30 days and then exposed to heat (35°C and 40% relative humidity) for 3 hr. The results showed that the immune function and anti‐oxidative enzyme activities in *L. lutea* in heat‐stressed environment were enhanced by chitosan supplementation, whereas oxidative damage of tissues and cells were alleviated. The results revealed that addition of 0.5% chitosan to the diet may be optimal, playing a key role in meeting the demands of captive‐bred *L. lutea* in high‐temperature environments. This may constitute a useful feeding strategy in accordance with the behavioural selection of wild *L. lutea*, and could effectively promote ex situ conservation.

## Graphical abstract

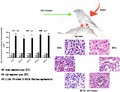

## 1 INTRODUCTION

The Red‐billed Leiothrix (*Leiothrix lutea*) is widely distributed in southern China. Due to its small and exquisite body shape, mildly sweet sound and aesthetic plumage, *L. lutea* is a favourite bird among aviculturists worldwide (Male et al., 1998). With the increasing trade of *L. lutea*, hunting pressure has dramatically increased and the amounts of wild *L. lutea* have drastically declined (Dai & Zhang, 2017). As a result of the Convention on the International Trade of Endangered Species of Fauna and Flora (CITES‐1997), the worldwide trade of wild‐caught *L. lutea* has been prohibited due to the depletion of native populations of this species. Therefore, captive‐bred *L. lutea* might play a key role in meeting the demands of trade and the recovery of wild populations of this species.

Meanwhile, a study presenting records over a 10‐year period of a captive colony of *L. lutea* showed that the main causes of bird loss include rearing management failures and age‐related disorders. Our previous study demonstrated that the breeding season of wild *L. lutea* stretches from April to September, with environmental temperatures of 14–20°C (Zhou et al., 2012). However, in southern China, especially in places where most *L. lutea* are distributed and raised, the temperature of the artificial feeding environment is higher than the above values, sometimes as high as 40°C. Heat stress could cause multiple physiological and metabolic changes in birds such as increased body temperature (Deyhim & Teeter, 1992), and enhanced reactive oxygen species (ROS) production and oxidative stress induction in cells (Flanagan et al., 1998; Lord‐Fontaine & Averill‐Bates, 2002).

In a previous field survey, we found that *L. lutea* forage on Coleoptera insects, which are rich in chitin for nestlings (Wang et al., 2015), indicating that such materials are required during their reproductive period (Cruz Cláudio et al., 2011). Chitosan is derived from chitin by deproteinization, demineralization and deacetylation. Many studies on poultry and swine have found that chitosan improves growth performance (Huang et al., 2005), enhances immune function (Xiao et al., 2013) and increases antioxidant (Niu et al., 2013), antimicrobial and hypocholesterolemic (Swiatkiewicz et al., 2015) properties. Hence, we hypothesized that physiological and biochemical changes in heat‐stressed birds could be alleviated by chitosan supplementation.

Therefore, the present study aimed to assess immune function indexes, antioxidant enzyme activities and histological changes in *L. lutea* exposed to acute heat stress, and to evaluate the effects of chitosan supplementation on these parameters.

## 2 METHODS

### 2.1 Birds, housing and feeding

The study was performed at the Key Laboratory of Southwest China Wildlife Resource Conservation (China West Normal University), Ministry of Education and met the guidelines approved by the institutional animal care and use committee. In the preparatory period, birds (adults from the Laboratory of Bird Breeding) were housed in large wire cages (4.8 m × 2.1 m × 2.4 m), with perches built of branches. Cages were decorated with *Cinnamomum japonicum*, *Rhapis gracilis* and other potted plants, with hay flakes laid on the floor near the window (e.g. *Bermuda* grass, fibrous roots of *Ficus microcarpa* and palm silk) to entertain the birds. Food containers, water boxes and tubs were placed in the open spaces. The tub depth was appropriate to prevent birds from drowning.

During the experiment, 80 birds (approximately 18 months old; average bodyweight, 17.95 ± 0.11 g) were selected from large wire cages and randomly divided into five experimental groups (*n* = 16 per group). Each treatment had four replicates, each containing four birds, which were housed in small wire cages (0.69 m × 0.41 m × 0.32 m). The pre‐feeding and feeding experiments lasted 7 days and 30 days respectively. All birds were maintained in the normal‐temperature environment at 21°C with 60% relative humidity in the pre‐feeding and feeding periods. At 37 days, birds in groups 2–5 were exposed to heat stress (35°C and 40% relative humidity) for 3 hr. Group 1 was kept at a constant normal temperature and represented the control group.

Diet preparation was based on nutrient requirements of the daily diet of the American quail for US NRC (Dale, 1994). The basal diet consisted of cereal seed mixture, soybean meal, fish meal and feed additives (Table 1). The cereal seed mixture consisted of maize, broken rice, sorghum, wheat bran, rice bran and flour. The animal protein mixture was made from fish meal. Chitosan was supplemented to the basal diet for the five groups in quantities of 0 (in the normal‐temperature environment), 0, 0.1%, 0.5% and 1% respectively. Chitosan (degree of deacetylation >90%, viscosity between 60 and 100 mPa.s) was provided by Jinan Haidebei Marine Bioengineering. The birds had free access to feed and water during the rearing period; food but not water was withdrawn during the 3‐hr experimental heat stress period.

**TABLE 1:** Composition and nutritional value of the basal diet

Dietary component	Percentage (%)	Nutrient level	
Corn	30	ME (MJ kg ^–1^)	12.06
Soybean	7	Crude protein (%)	20.02
Rice	12.8	Ca (%)	0.81
Soybean meal	22	P (%)	0.59
Wheat bran	4	Lysine (%)	1.11
Flour	12	Methionine (%)	0.45
Rice bran	4		
Fish meal	6		
Methionine	0.1		
CaCO_3_	1.3		
Salt	0.2		
1% premix a	0.6		
Total	100		

Note

1% premix provided per kilogram of complete feed: 12 mg retinol, 2.5 mg pyridoxine, 0.02 mg cholecalciferol, 20 mg tocopherol, 50 mg nicotinic acid, 2 mg menadione, 12 mg pantothenic acid, 12μg cyanocobalamin, 6 mg riboflavin, 0.30 mg biotin, 1.10 mg folic acid, 1,500 mg choline, 100 mg Fe, 25 mg Zn, 6 mg Cu, 90 mg Mn, 0.2 mg Se, 0.3 mg I and 0.05 mg Mg.

### 2.2 Sample collection and preparation

After heat exposure, 40 birds (two birds per pen, eight birds in each group) were randomly selected for blood collection from a wing vein with a heparinized syringe. Blood specimens were centrifuged at 4,000 rpm for 10 min at 4°C, and the resulting plasma was divided into two and stored at −20°C for further analysis. After blood sampling, all birds were euthanized, and liver and chest muscle samples were collected immediately after dissection, washed with ice‐cold saline (0.9%), flash frozen in liquid nitrogen and kept at −80°C until analysis. All samples were run in the same assay to avoid inter‐assay variability. The reagents used in this study were obtained from Sigma‐Aldrich Chemical Co. (St Louis, MO, USA) unless otherwise specified.

Plasma immunoglobin G, A and M concentrations were measured with a commercial kit (ERKN Inc., China) by enzyme‐linked immunosorbent assay (ELISA). Glutathione peroxidase (GSH‐Px), superoxide dismutase (SOD), catalase (CAT) and malondialdehyde (MDA) were measured with specific commercial kits (Nanjing Jiancheng Bioengineering Institute, China) by ultraviolet spectrophotometry.

Tissue samples were homogenized in nine volumes of 10 mmol sodium phosphate buffer (pH 7.4) containing 1.15% potassium chloride. After centrifugation of the homogenates at 400g for 10 min at 4°C, the supernatants were used for further measurements. GSH‐Px, SOD, CAT and MDA activities were determined as described for plasma samples.

Histopathological examination was performed on 20 birds (one bird per pen, four birds per group) that were euthanized after heat exposure. Liver and spleen samples were collected immediately after dissection, fixed with 4% paraformaldehyde (PFA) and paraffin embedded. Thin sections (5 μm) were mounted on glass, and stained with haematoxylin and eosin (HE). The histological structures were observed and imaged with a digital camera (Nikon, eclipse 50i, Japan).

### 2.3 Statistical analysis

All data were assessed by ANOVA with SPSS v 19.0 (SPSS Inc., Chicago, USA). The cage was the experimental unit. Data are mean ± SE. Specific pre‐planned backgrounds were used to assess the effect of supplemental chitosan. *p* < .05 and *p* < .01 were considered significant and very significant respectively.

## 3 RESULTS

### 3.1 Plasma immunoglobulin concentrations

**FIGURE 1:**
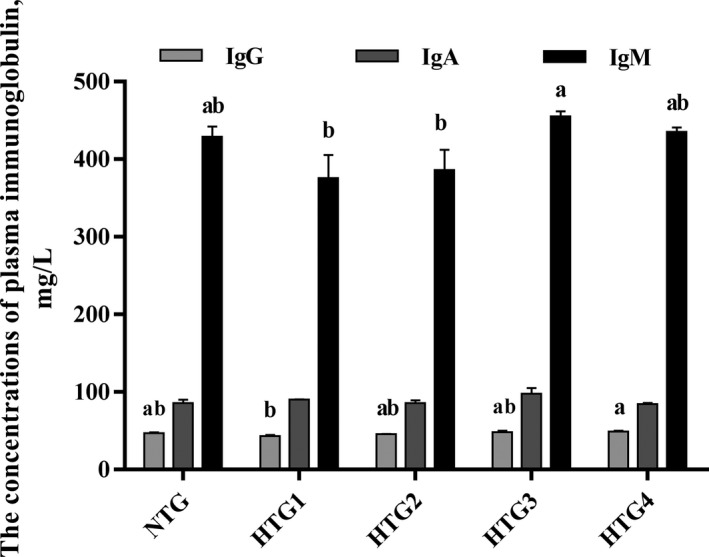
Effects of chitosan supplementation on plasma immune globulin levels in heat‐stressed *Leiothrix lutea* (mean ± *SD*). NTG, normal‐temperature group (21°C); HTG, high‐temperature group (35°C); HTG 1, high‐temperature group without chitosan supplementation; HTG 2, high‐temperature group with 0.1% chitosan supplementation; HTG 3, high‐temperature group with 0.5% chitosan supplementation; HTG 4, high‐temperature group with 1% chitosan supplementation; ^a,b^, means within the same index without a common superscript lowercase differed significantly (*p* < .05)

Compared with the normal‐temperature group, plasma IgG and IgM concentrations were decreased in high‐temperature groups (Figure 1). However, plasma IgG and IgM levels were increased after chitosan supplementation in the HTGs. Compared with the HTGs without chitosan supplementation (HTG 1), IgG concentrations were increased by 12.7% (P < .05) in the HTG with 1% chitosan supplementation (HTG 4); IgM concentrations were increased by 21.07% (P < .05) in the HTG with 0.5% chitosan supplementation (HTG 3).

### 3.2 Activities of antioxidant enzymes and tissue peroxidation status in plasma and tissues

**FIGURE 2:**
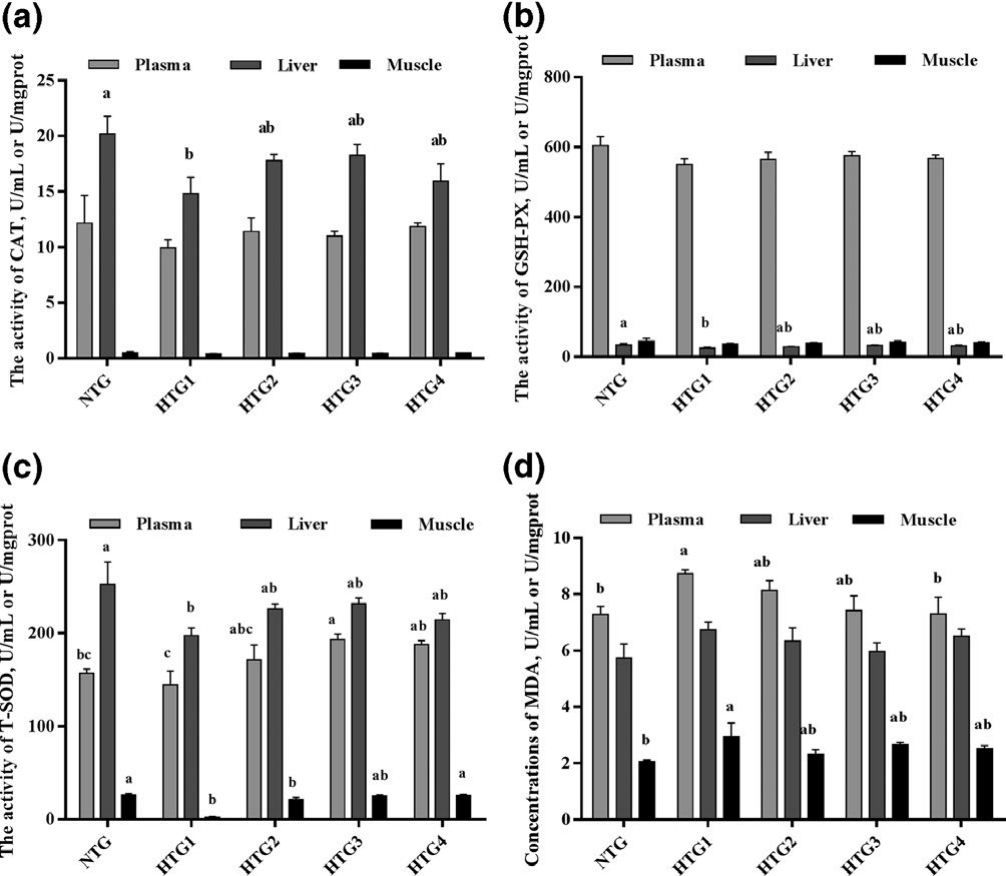
Effects of chitosan on the activities of CAT (A), GSH‐Px (D), T‐SOD (C) and MDA (D) levels in tissues of heat‐stressed *Leiothrix lutea* (mean ± *SD*). CAT, catalase; GSH‐Px, glutathione peroxidase; T‐SOD, total superoxide dismutase; MDA, malondialdehyde; NTG, normal‐temperature group (21°C); HTG, high‐temperature group (35°C); HTG 1, high‐temperature group without chitosan supplementation; HTG 2, high‐temperature group with 0.1% chitosan supplementation; HTG 3, high‐temperature group with 0.5% chitosan supplementation; HTG 4, high‐temperature group with 1% chitosan supplementation; ^a,b^, means within the same index without a common superscript lowercase differed significantly (*p* < .05)

The activities of antioxidant enzymes in plasma and tissue samples in the HTGs are shown in Figure 2. Compared with the NTG, the HTGs showed decreased antioxidant enzyme activities in plasma and tissues, except plasma activities of SOD in HTG 2, HTG 3 and HTG 4. In the HTGs, with increasing chitosan supplementation, the activities of antioxidant enzymes showed an increase, but were not significant. Compared with HTG 1, plasma SOD activity in HTG 3 and muscle SOD activity in HTG 4 were increased (*p* < .05). GSH‐Px activities in HTG 3 for all tissues and CAT activity in the liver in HTG 3 showed an increase, but was not significant (*p* > .05).

In plasma and muscle samples, MDA concentrations were increased (*p* < .05) by acute stress, and this effect was alleviated by chitosan supplementation. Compared with HTG 1, plasma MDA concentrations in HTG 4 were decreased (*p* < .05). In the liver, no differences were observed in MDA concentrations between the NTG and HTGs.

### 3.3 Effect of chitosan supplementation on histological changes in the spleen and liver in acute heat‐stressed *L. lutea*

**FIGURE 3:**
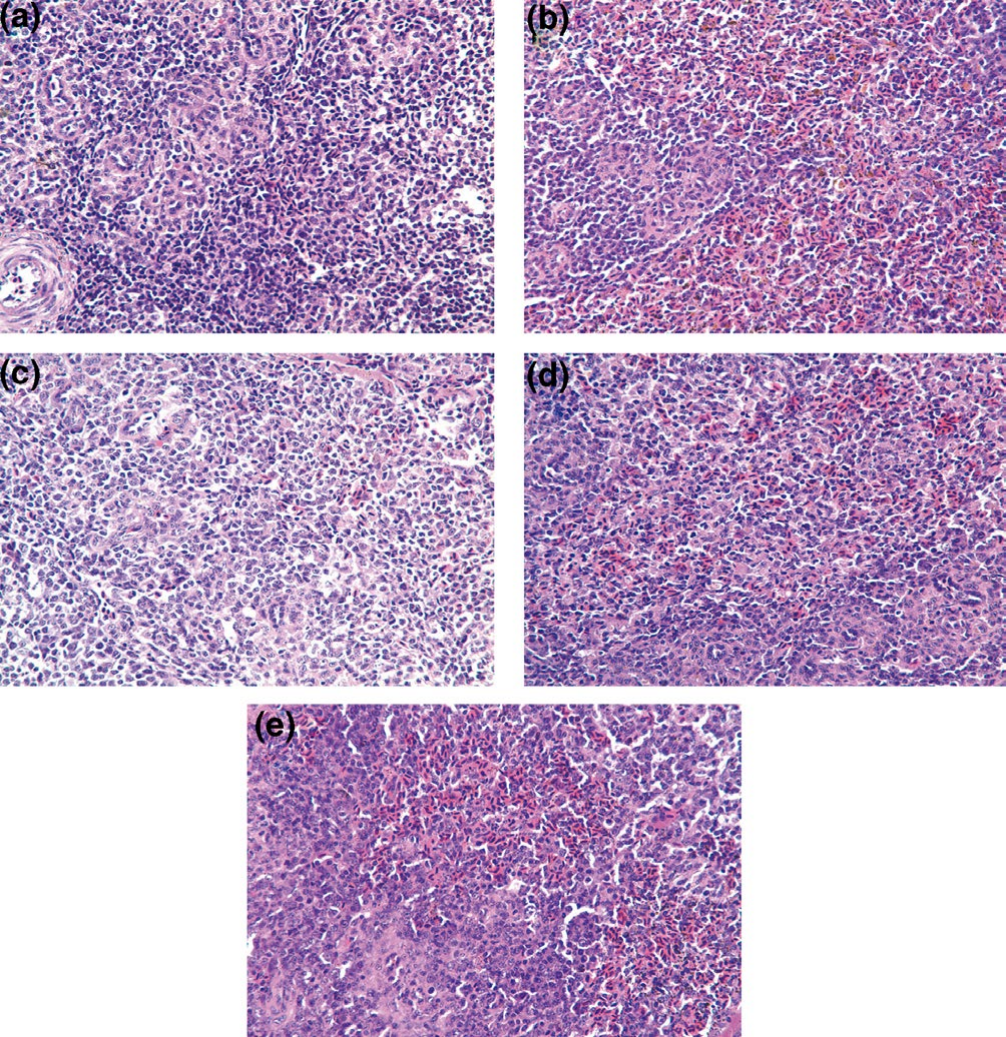
Histopathological changes in spleen of *Leiothrix lutea* (HE × 400). (a) Normal‐temperature group (21°C), NTG; (b) HTG 1, high‐temperature group without chitosan supplementation; (c) HTG 2, high‐temperature group with 0.1% chitosan supplementation; (d) HTG 3, high‐temperature group with 0.5% chitosan supplementation; (e) HTG 4, high‐temperature group with 1% chitosan supplementation

Histological changes in the spleen of *L. lutea* are shown in Figure 3. The boundary between the splenic red pulp and the white medulla was clear, with a moderate proportion. The *acini lienalis* was clearly visible, and there was no overt pathological change in the spleen of NTG *L. lutea* (Figure 3a). In HTG 1, the central and sheath arteries, and the red pulp region of the spleen showed different degrees of dilation. The sinus was full of red blood cells, and the *acini lienalis* showed atrophy (Figure 3b). In other *L. lutea* exposed to high temperature and treated with chitosan, mild hyperaemia was found in the red pulp region, and histological changes in the spleen were less pronounced than that of HTG 1 (Figure 3c‐e).

**FIGURE 4:**
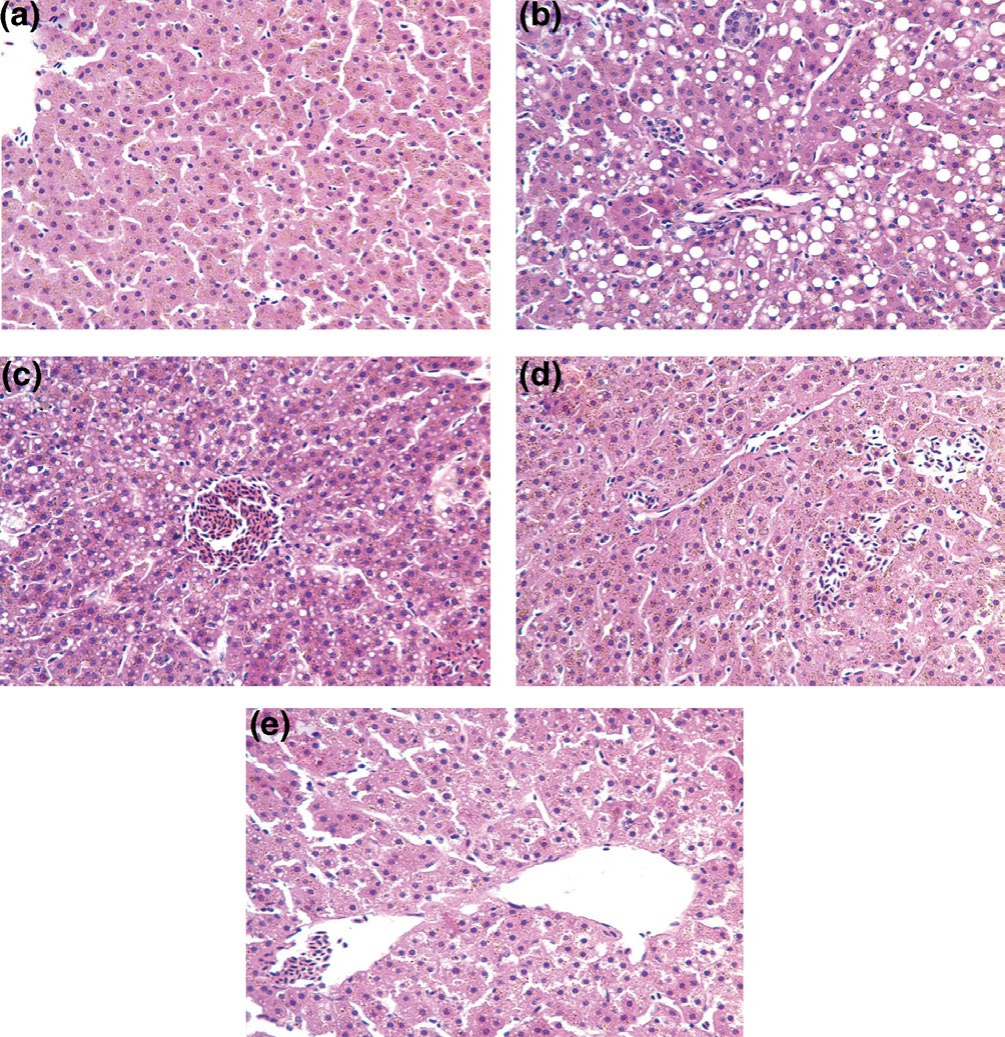
Histopathological changes in the liver of *Leiothrix lutea* (HE × 400). (a) Normal‐temperature group (21°C), NTG; (b) HTG1, high‐temperature group without chitosan supplementation; (c) HTG 2, high‐temperature group with 0.1% chitosan supplementation; (d) HTG 3, high‐temperature group with 0.5% chitosan supplementation; (e) HTG 4, high‐temperature group with 1% chitosan supplementation

Histological changes in the liver of *L. lutea* are shown in Figure 4. The structure of the hepatic cord was clear, and hepatocyte cytoplasm was homogenous, with no hyperaemia or obvious pathological changes in the liver of NTG birds (Figure 4a). Meanwhile, the volume of hepatocytes was increased, with the cytoplasm showing large amounts of near‐circular vacuoles, resulting in a loose arrangement of the cytoplasmic structure in hepatocytes, whose swelling compressed liver sinusoids in HTG 1 birds (Figure 4b). There were multiple fat droplets in the hepatocyte cytoplasm of HTG 2 birds, and the volume of adipose hollow space was smaller compared with those of the HTG (Figure 4c). The microscopic structures of liver tissues in HTG 3 and HTG 4 birds were similar to those of NTG birds (Figure 4d,e).

## 4 DISCUSSION

### 4.1 Acute heat stress model in *L. lutea*

Our study observed that acute heat stress may lead to changes in antioxidant capacity and histological changes in birds. It is generally accepted that heat stress plays a role in the animal's immune system through the hypothalamus‐pituitary‐adrenal axis, stimulating the hypothalamus and pituitary gland to secrete cortisol, which acts on the adrenal cortex and promotes the generation of glucocorticoids that inhibit immunoglobin synthesis and alter immune function (Donker et al., 1990). The above immunoglobulin findings in the HTG corroborated previous studies assessing heat stress in calves (Kelley, 1980), further confirming that heat stress inhibits immune function in animals.

ROS are continuously generated in cells by several oxidative enzymes and by dismutation of the superoxide anion formed by electron leakage during mitochondrial respiration (Fridovich, 1978). Under normal circumstances, to protect the body from damage, the generation, utilization and elimination of free radicals in the organism should maintain a dynamic balance. Studies of mammalians showed that hyperthermia enhances ROS production (Flanagan et al., 1998; Hall et al., 1994), decreases the activities of anti‐oxidative enzymes and induces lipid peroxidation (Lin et al., 2000). In the present study, anti‐oxidative enzymes such as SOD, GSH‐Px and CAT activities as well as oxidation products concentrations such as MDA were employed to reflect responses of enzymatic systems and lipid peroxidation in tissues respectively. Compared with the NTG, the HTGs showed significantly decreased SOD activities in the muscle and reduced amounts of the three detected enzymes in the liver; meanwhile, the concentrations of MDA in plasma, liver and muscle samples from the HTGs were significantly increased. The different responses of anti‐oxidative enzyme activities and oxidation product concentrations (MDA) concentrations in the plasma, liver and muscle suggest tissue specificity. These results corroborate previous findings that anti‐oxidative enzyme activity in the liver could reflect the status of heat stress (Lin et al., 2000; Morrison et al., 2005).

As the rates of many chemical and biochemical reactions increase with temperature, it is thus likely that increased body temperature would enhance ROS generation via accelerated metabolic reactions and cause oxidative damage in cells and tissues (Lin et al., 2004). The current study suggested that heat stress could further aggravate splenic and hepatic damage and induce bleeding in tissues, as well as cell atrophy and vacuole degeneration.

### 4.2 Acute heat stress is alleviated by chitosan supplementation in *L. lutea*

Chitosan is an acetylated amino‐polysaccharide (Muzzarelli Riccardo, 1977). An in vivo study found that chitosan reduces the concentrations of oxidative stress indicators in the systemic circulation, with direct antioxidant activities (Donker et al., 1990). In this study, chitosan supplementation to the diet of artificial breeding *L. lutea* exposed to high‐temperature stress increased immunoglobin concentrations and the activities of anti‐oxidative enzymes in tissues, enhanced antioxidant abilities and reduced ROS damage in cells. This is supported by studies in cattle, where the addition of chitosan to the basic diet of milk cows increases the concentrations of plasma IgG, IgM and IgA in milk (Liu et al., 2007). These findings indicate that chitosan could be used as immune enhancer in animals. The possible mechanism by which chitosan protects from heat stress may be related to the mitogen‐activated protein kinases (MAPK) signalling pathway (Li et al., 2014), nuclear factor‐κB (NF‐κB) signalling (Kang et al., 2011), immune‐modulatory factors and lipid metabolism, but requires further investigation.

In this study, chitosan showed a certain anti‐thermal stress effect only at concentrations above 0.5% in *L. lutea's* diet, consistent with previous research results (Shi et al., 2005). In the latter report, broiler chickens were fed diets containing 0.02%–0.50% chitosan, and antibody titres and lymphocyte transformation rates were increased in the 0.1%–0.5% chitosan groups. These findings suggest that an appropriate amount of supplemented chitosan may be a normal requirement for birds, and is especially necessary in stressful conditions.

This study demonstrated that immune function and anti‐oxidative enzyme activities in *L. lutea* during heat stress could be enhanced by chitosan supplementation, alleviating oxidative damage in tissues and cells. The above results revealed that adding 0.5% chitosan to the diet may be optimal and could help meet the demands of captive‐bred *L. lutea* in high‐temperature environments. This may constitute a useful feeding strategy in accordance with the behavioural selection of wild *L. lutea*, and could effectively promote ex situ conservation.

## CONFLICT OF INTERESTS

There are no conflicts of interest.

## AUTHOR CONTRIBUTION

Yi Dai: Conceptualization; Formal analysis; Visualization; Writing‐original draft; Writing‐review & editing. Ming‐qiang Zhou: Data curation; Investigation; Methodology. Yun‐qian He: Investigation; Methodology. Xi Peng: Conceptualization; Data curation; Methodology; Visualization. Shi‐bin Yuan: Conceptualization; Data curation; Investigation; Methodology; Project administration; Resources; Software; Supervision; Visualization.

### PEER REVIEW

The peer review history for this article is available at https://publons.com/publon/10.1002/vms3.387.
